# Supplementary figures and images for: Co-expression of bovine leukemia virus and bovine foamy virus-derived miRNAs in naturally infected cattle
Source: Microbiol Spectr. 2025 Sep 11;13(10):e01755-25. doi: 10.1128/spectrum.01755-25 (PMC12502788; doi:10.1128/spectrum.01755-25)

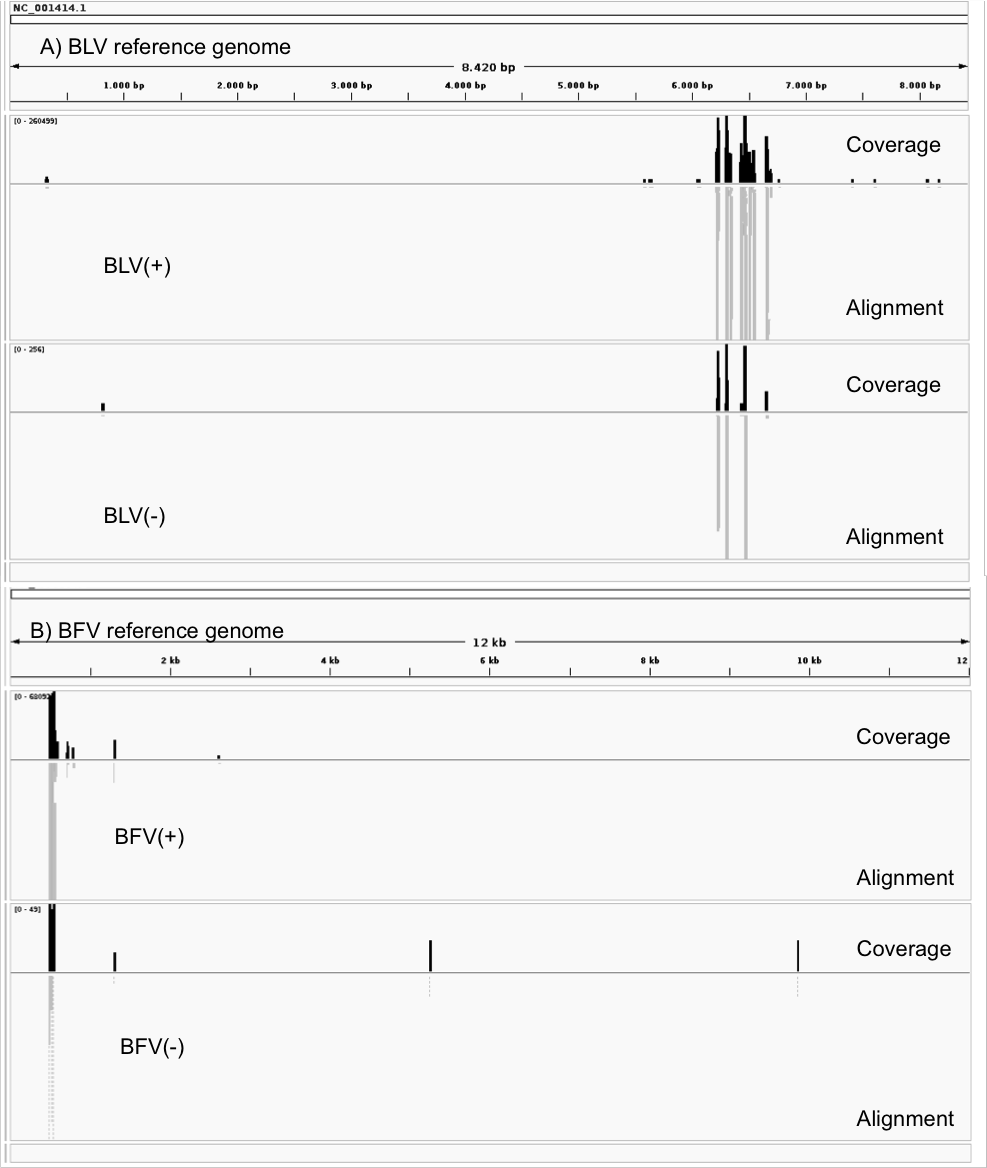

Supplement: Fig. S1 — Integrative genomics viewer visualizations of sequencing reads alignment from BLV(+) and BLV(−) samples to BLV and BFV reference genomes. [file spectrum.01755-25-s0001.tiff]

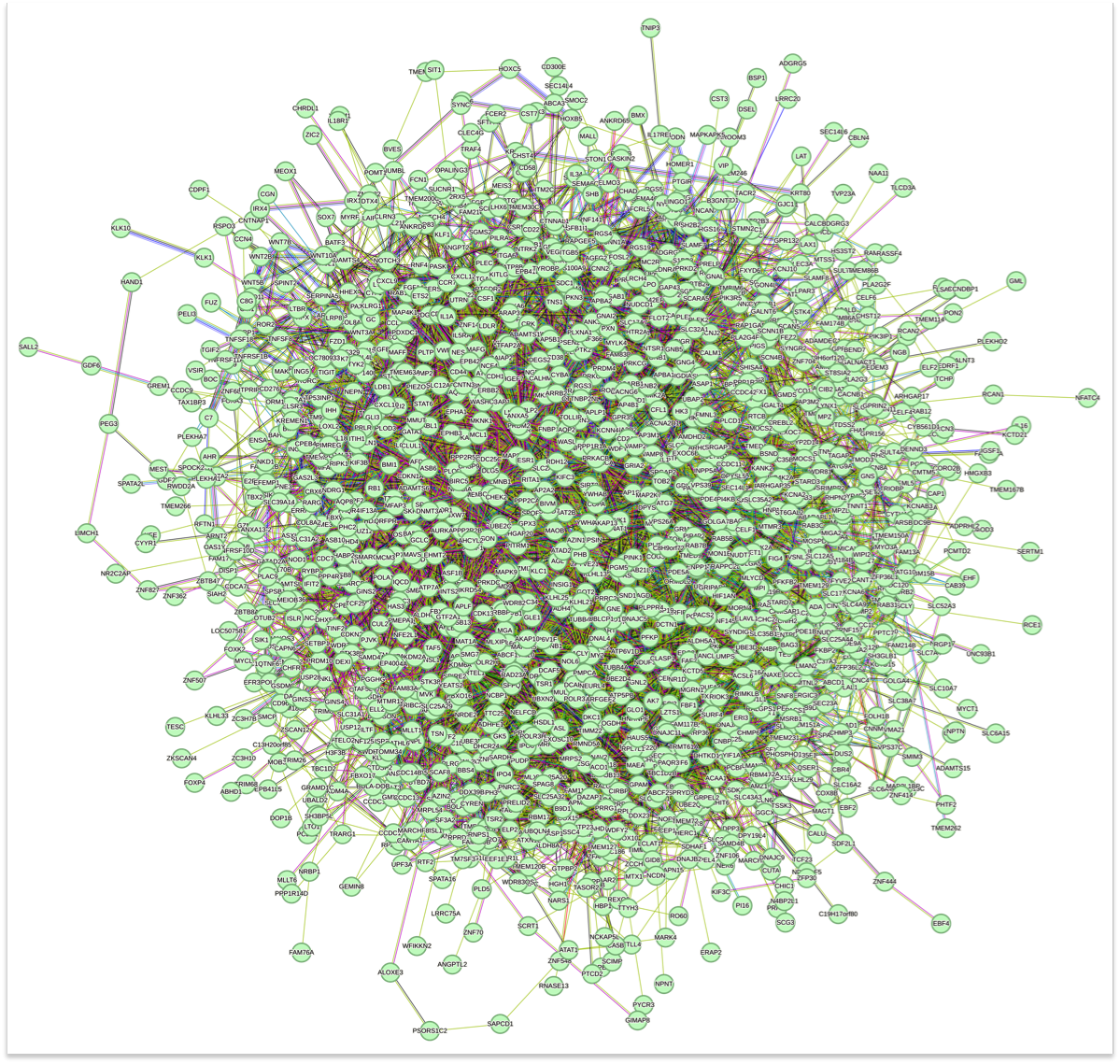

Supplement: Fig. S2 — PPI network for predicted gene targets of BTA-miRNAs-DE, BLV-miRNAs, and BFV-miRNAs. [file spectrum.01755-25-s0002.tiff]
