# Supplementary material for: Co-expression of bovine leukemia virus and bovine foamy virus-derived miRNAs in naturally infected cattle
Source: Microbiol Spectr. 2025 Sep 11;13(10):e01755-25. doi: 10.1128/spectrum.01755-25 (PMC12502788; doi:10.1128/spectrum.01755-25)
Supplement: Supplemental material — Legends for all supplemental material. [file spectrum.01755-25-s0003.docx]

**Supplementary Material**

Supplementary Figure S1. Integrative genomics viewer visualizations of sequencing reads aligment from BLV(+) and BLV(–) samples to Bovine leukemia virus (BLV) and Bovine foamy virus (BFV) reference genomes. The black bar represents the read coverage (log scale) across the entire length of BLV (acc. NC_001414.1) (A) and BFV (acc. NC_001831.1) (B). The coverage range for BLV(+) samples aligned to BLV genome (length 8,419 bp) was 0 to 260,499, while BLV(–) samples ranged (0 to 256). For the BFV genome (length 12,002 bp), the BLV(+) samples coverage ranged from 0 to 68,092, while BLV(–) coverage ranged from 0 to 49. Viral miRNAs aligned to specific genomic regions: BLV: 6620 – 6700 pb, BFV: 315-525 pb. For BFV, only aligments over the 5´ long terminal repeat (LTR) are showed.

Supplementary Figure S2. Protein-Protein Interaction (PPI) Network for predicted gene targets of BTA-miRNAs-DE, BLV-miRNAs and BFV-miRNAs. Nodes represent proteins encoded by predicted miRNA gene target. Edges represent protein-protein interactions identified through STRINGdb evidence, including: known interactions (curated databases and/or experimentally validated), predicted interactions (gene fusions, gene co-occurrence), text mining, co-expression, and protein homology. Number of nodes: 1,017; Number of edges: 2,644; Average node degree: 5.2 and PPI enrichment p-value: 2.3e^-10^.

Supplementary Table S1. Number of reads mapped to mature miRNAs references for bovine, BLV, BFV, BoHV1 and BoHV5.

Supplementary Table S2. miRNAs differential expression analysis.

Supplementary Table S3. Predicted target genes for BLV-miRNAs, BFV-miRNAs and BTA-miRNAs-DE.

Supplementary Table S4. Gene Ontology categories (BP, MF and CC), KEGG and Reactome metabolic pathways significantly overrepresented in main protein-protein interaction network and Clusters C1-C3.
